# Deception and Shopping Behavior Among Current Cigarette Smokers: A Web-Based, Randomized Virtual Shopping Experiment

**DOI:** 10.2196/10468

**Published:** 2018-06-29

**Authors:** Lauren McCarl Dutra, James Nonnemaker, Nathaniel Taylor, Annice E Kim

**Affiliations:** ^1^ Center for Health Policy Science and Tobacco Research RTI International Berkeley, CA United States; ^2^ Center for Health Policy Science and Tobacco Research RTI International Research Triangle Park, NC United States

**Keywords:** online shopping, deception, social and behavioral sciences, consumer behavior, smokers

## Abstract

**Background:**

Virtual stores can be used to identify influences on consumer shopping behavior. Deception is one technique that may be used to attempt to increase the realism of virtual stores.

**Objective:**

The objective of the experiment was to test whether the purchasing behavior of participants in a virtual shopping task varied based on whether they were told that they would receive the products they selected in a virtual convenience store (a form of deception) or not.

**Methods:**

We recruited a US national sample of 402 adult current smokers by email from an online panel of survey participants. They completed a fully automated randomized virtual shopping experiment with a US $15 or US $20 budget in a Web-based virtual convenience store. We told a random half of participants that they would receive the products they chose in the virtual store or the cash equivalent (intervention condition), and the other random half simply to conduct a shopping task (control condition). We tested for differences in demographics, tobacco use behaviors, and in-store purchases (outcome variable, assessed by questionnaire) by experimental condition.

**Results:**

The characteristics of the participants (398/402, 99.0% with complete data) were comparable across conditions except that the intervention group contained slightly more female participants (103/197, 52.3%) than the control group (84/201, 41.8%; *P*=.04). We did not find any other significant differences in any other demographic variables or tobacco use, or in virtual store shopping behaviors, including purchasing any tobacco (*P*=.44); purchasing cigarettes (*P*=.16), e-cigarettes (*P*=.54), cigars (*P*=.98), or smokeless tobacco (*P*=.72); amount spent overall (*P*=.63) or on tobacco (*P*=.66); percentage of budget spent overall (*P*=.84) or on tobacco (*P*=.74); number of total items (*P*=.64) and tobacco items purchased (*P*=.54); or total time spent in the store (*P*=.07).

**Conclusions:**

We found that telling participants that they will receive the products they select in a virtual store did not influence their purchases. This finding suggests that deception may not affect consumer behavior and, as a result, may not be necessary in virtual shopping experiments.

## Introduction

Virtual reality retail stores enable researchers to examine the real-time impact of changes in the retail environment on consumer behavior. Virtual store experiments can be used to understand the effects of changes such as store layout, product placement, advertising, and the presence of public health mass media campaigns on behaviors such as the type and number of products purchased and time spent in the store. Virtual stores provide a better method than static images on a computer screen of testing changes in the shopping environment [1] and offer the option of ensuring the feasibility of a business concept with a smaller investment of time and resources than would be needed for brick-and-mortar stores. Virtual supermarkets have also been used by researchers to solve problems in completing daily living skills among individuals with mental health problems and to assess cognitive functioning [2-4].

One persistent question in virtual store research is the degree to which the products that consumers select in the virtual store reflect the products they would select in a brick-and-mortar store or the products they actually want to purchase. A few studies have validated virtual store purchases with other shopping scenarios and have produced promising results. One such experiment used participant-provided receipts from supermarket purchases to validate virtual store purchases and found high levels of consistency between virtual and actual purchases [5]. Similarly, Desmet et al [6] found similar attitudes toward goods regardless of whether the good was viewed in a virtual or in a real store environment. However, purchase time, visual recall, and brand loyalty varied by store type. Similarly, the behaviors of 5 patients with schizophrenia who were completing shopping tasks in a virtual supermarket were comparable with their behaviors in real-life grocery stores [2]. van Herpen et al [1] found some differences between a virtual store and a physical store. However, shopping behavior was more similar between the virtual and physical stores than between static images of the virtual store and physical stores.

One aspect of research on virtual store experiments that remains unexplored is whether using deception in the instructions for the virtual store may improve the realistic nature of the virtual store (make virtual shopping experiences more closely resemble actual shopping experiences). Deception is a commonly used [7-9] (and sometimes necessary [10]) experimental method in marketing and consumer behavior research, psychology, and economics; however, its utility has never been tested in a virtual shopping experiment. We created a virtual convenience store, iShoppe, to understand the impact of the store environment on purchases, particularly tobacco product purchases. Previous experiments with iShoppe have informed research on the tobacco retail environment. For example, we found differences in attempts to purchase tobacco products based on whether a display of tobacco products was visible (open) or enclosed in a cabinet [11,12].

The objective of the experiment was to test whether the purchasing behavior of participants in a virtual shopping task varied based on whether they were told that they would receive the products they selected in a virtual convenience store (a form of deception) or not. We varied the virtual store instructions for iShoppe to include deception or not. We hypothesized that we would find differences in virtual store shopping behavior based on whether the store instructions included deception or not.

## Methods

### Participants

Research Triangle Institute (RTI) International’s institutional review board approved all study procedures. Between May 4 and May 11, 2015 (the time necessary for the quota of 400 participants to be reached), Lightspeed Online Research, LLC (Warren, NJ, USA), a market research company that provides data collection services for consumer research studies, recruited an online (invitation-only or closed) sample of adult current cigarette smokers living in the United States from their existing global panel of more than 5.5 million survey participants Lightspeed recruits panel participants through email, internet ad banners, social media, e-newsletters, and referrals from recruiting partners.

Inclusion criteria for participation in the online-only randomized experiment were reporting living in one of the states in the United States, being the same age as or older than the tobacco minimum legal sales age for their state (18-21 years of age or older), and currently smoking cigarettes some days or every day (internet and computer literacy were also de facto inclusion criteria because the study was conducted online only). Exclusion criteria were not living in the United States, being under the tobacco minimum legal sales age for their state, and currently smoking cigarettes rarely or never.

### Study Procedures

Potential participants were invited by email to complete an online screener (6 items on 6 pages) to determine their eligibility to participate in the voluntary study: “GlobalTestMarket is looking for your opinion. Don’t miss out on being rewarded for sharing your opinions. Complete this survey today!” The email also contained a survey number, an offer of 100 “market points” (approximately US $5 worth of points that could be redeemed for products and services from several corporate sponsors) for survey completion, and the URL for the screener. If participants met the inclusion criteria, they were provided a unique participant identifier (no identifying information was collected) and completed the informed consent. The consent invited them to click a checkbox to agree to complete the virtual shopping task in RTI International’s iShoppe (developed in 2011 by a team of graphic designers at RTI International and software programmers, as part of a Robert Wood Johnson Foundation project in Research Triangle Park, NC, USA, using Unity 3D software; Multimedia Appendix 1) and, immediately afterward, a survey. In addition to the purpose of the study, the consent form included the number of participants recruited, risks and benefits of the study, information about incentives, confidentiality of the participants’ responses, efforts to protect the participants and their privacy, information about the Unity 3D software program that must be downloaded as part of the study, and contact information for the project director and institutional review board.

After consenting, participants were randomly assigned to the intervention (deception) condition or the control (no deception) condition in a one-to-one (parallel) ratio (using a random number generator built into the software). All participants were aware that they were participating in a research study, but they were blinded to experimental condition (researchers analyzing the resulting data were not blinded to condition). After providing consent, participants were provided a link to the virtual store experiment, which began by providing directions that participants could purchase anything in the store (within a budget) and that, when done, they should click on the virtual clerk, who would help them complete their purchases. All participants had a budget of either US $15 or US $20. We assigned the higher budget to participants living in areas with higher excise taxes on cigarettes to ensure that all participants could afford to purchase tobacco products in the virtual store. In the intervention condition, we also instructed participants that, after completing the virtual shopping task, they would receive the products they selected from the virtual store by mail or, if the products were unavailable, the cash equivalent. We gave participants in the control condition no additional instructions. No alcohol products were available for purchase in the virtual store because some participants were under 21 years of age.

After completing the virtual shopping task, participants completed a short survey assessing their demographics and tobacco use (51 items on 51 pages). We developed all surveys (screener and postexperiment survey) based on literature reviews and, when possible, by using and modifying items from existing surveys. Surveys were tested by study staff and non–study staff employed by RTI International before fielding. Surveys contained skip patterns to reduce participant burden, but the order of the items was not random. Participants were required to complete all survey items (they could respond “prefer not to answer” or “don’t know”) before advancing to the next page or submitting the surveys. We analyzed only surveys with complete responses. Participants were able to log out and back in to the survey to accommodate participants unable to complete the experiment in one sitting. Speed checks were used to identify participants who completed the survey too quickly; however, no surveys were excluded for this reason.

At the end of the survey, during the debriefing, all participants were instructed that they would receive a Visa e-gift card for US $20 (in addition to the Lightspeed incentive). Participants in the intervention condition were informed that they would receive the gift card in place of receiving the items they selected in the store by mail.

### Variables

#### Descriptive Variables

We examined demographic and tobacco use variables across the intervention and control groups to characterize the sample and ensure that randomization was complete. Demographic variables, all of which were categorical, were age (≤34 years, 35-54 years, or ≥55 years of age); sex (male or female); race/ethnicity (non-Hispanic white, non-Hispanic black, Hispanic, or non-Hispanic other); education (some high school or less, high school graduate or General Education Development, some college, or college graduate or beyond, such as postgraduate or professional degree); and annual household income (≤$25,000, $25,000-$49,999, $50,000-$74,999, or ≥$75,000 annually, all in US $). Categorical tobacco use variables were number of days smoked in the past 30 (none, 1-2 days, 3-5 days, 6-9 days, 10-19 days, 20-29 days, or 30 days); ever daily smoking (yes or no); the last time the participant smoked (within the last hour, 1-2 hours ago, 2-5 hours ago, 5-10 hours ago, within the past day, or 2 days ago or longer); how soon after waking the participant usually smoked his or her first cigarette (≤5 minutes, 6-30 minutes, 31-60 minutes, or after 60 minutes); current smokeless tobacco use (yes or no); current use of cigars, cigarillos, or little cigars (yes or no); stopping smoking for 1 day or more in the past 12 months (yes or no); desire to quit smoking (not at all, a little, somewhat, or a lot); and planning to stop smoking in the next 30 days (yes or no). The only continuous variable was mean number of cigarettes per day smoked in the past 30 days. In addition, we examined the percentage of participants in the deception condition who reported that they understood that they might get all or some of the products that they selected in the store and that they might get a gift card in place of those products. Participants in the no deception condition were not asked this question.

#### Outcome Variables

The primary outcome variable, which was self-assessed online, was clicking to purchase any tobacco products. Secondary outcome variables were clicking to purchase cigarettes, e-cigarettes, cigars, or smokeless tobacco products (coded as 1 or more versus 0, the reference category), number of total items and tobacco product items purchased, dollars and percentage of the budget spent in total and on tobacco products only, and amount of time spent in the store.

#### Independent Variable

The independent variable was condition (intervention or control, reference).

### Analysis

We used *t* tests for continuous variables and chi-square tests for categorical variables (or Fisher exact test as necessary for small cell sizes) to test for differences in demographics, tobacco use variables, and in-store behavior by condition. All analyses were completed at RTI International (Berkeley, CA office) in Stata 14 (StataCorp LLC), and significance was determined as a value of *P*<.05.

## Results

### Participants

Lightspeed contacted via email over 30,000 (32,588) members, of whom 2607 (8.00%) visited the screener site [13]. Of these, 402 (15.42%) completed the survey, 286 (10.97%) screened out, 9 (0.35%) attempted to complete the survey after the quota for participation had been reached, and 1910 (73.26%) either did not complete the screener or screened in but chose not to participate or did not complete the survey.

**Table 1:** Virtual store behaviors by experimental condition^a^ among participants who completed a virtual shopping experiment (n=398).

Outcomes		Experimental group		Total (n=398)	*P* value
		Control (n=201)	Intervention (n=197)		
**Primary outcome (purchases), n (%)**					
	Any tobacco	124 (61.7)	114 (57.9)	238 (59.8)	.44
	Cigarettes	115 (57.2)	99 (50.3)	214 (53.8)	.16
	e-Cigarettes	14 (7.0)	17 (8.6)	31 (7.8)	.54
	Cigars	3 (1.5)	3 (1.5)	6 (1.5)	.98
	Smokeless tobacco	4 (2.0)	3 (1.5)	7 (1.8)	.72
**Secondary outcomes, mean (SD)**					
	Amount spent in US $	14.6 (3.9)	14.4 (3.5)	14.5 (3.7)	.63
	Percentage of budget spent	86.9 (19.0)	87.3 (18.5)	87.1 (1.9)	.84
	Amount spent on tobacco products in US $	5.4 (5.2)	5.1 (5.2)	5.2 (5.2)	.66
	Percentage of budget spent on tobacco products	31.9 (30.0)	30.9 (30.7)	31.4 (30.3)	.74
	Number of items purchased	5.9 (3.2)	5.8 (2.7)	5.9 (3.0)	.64
	Number of tobacco product items purchased	0.9 (0.9)	0.8 (0.9)	0.9 (0.9)	.54
	Total time in store (minutes)	5.0 (3.9)	5.7 (3.9)	5.3 (3.9)	.07

^a^Participants in the intervention (deception) condition were told that they would receive the products that they selected in the virtual store by mail (or the cash equivalent if the products were unavailable). Participants in the control (no deception) condition were told they would receive the cash equivalent of the products they selected.

Only 46.99% (1225/2607) of those who clicked on the link finished the first page of the screener. Of the 402 participants, 398 (99.0%) provided complete data (Multimedia Appendix 2 shows the flow diagram of the selection process). Reporting of the methods and results of this study complies with the CONSORT-EHEALTH guidelines (Multimedia Appendix 3) [14].

### Descriptive Statistics

Overall, participants were predominantly white with high levels of education and income (Multimedia Appendix 4). More than half were daily smokers, and almost all had ever smoked daily. Almost half had smoked within the last hour, more than half usually smoked within a half hour of waking, and about one-quarter reported current use of smokeless tobacco. One-third of participants reported current use of cigars, cigarillos, or little cigars. About one-third had made a quit attempt in the past 12 months, one-third wanted to quit smoking “a lot,” and 68.3% (272/398) planned to stop smoking in the next 30 days. Mean number of cigarettes smoked per day was 16.0 (SD 14.1). Among participants in the deception condition, 77.2% (152/197) reported that they understood that they would receive the products they selected (or some of them) or a gift card.

### Analysis

The control condition had slightly fewer female participants than the intervention condition (*P*=.04). There were no other significant differences in demographics or tobacco use behaviors between conditions.

### Results of Analysis

The percentage of participants who purchased any tobacco products did not vary by condition (*P*=.44; Table 1). Also, there was no difference by condition in the percentage of participants who purchased specific types of tobacco products, including cigarettes (*P*=.16), e-cigarettes (*P*=.54), cigars (*P*=.98), and smokeless tobacco (*P*=.72). The total amount of money spent (*P*=.63), percentage of budget spent (*P*=.84), and number of items purchased (*P*=.64) did not vary by intervention condition. Similarly, the amount spent on tobacco products (*P*=.66), percentage of budget spent on tobacco products (*P*=.74), and number of tobacco product items purchased (*P*=.54) did not vary by condition. Total time spent in the store was slightly longer in the intervention condition than in the control condition, but this difference was only marginally significant (*P*=.07).

## Discussion

### Principal Findings

We found no significant differences in the purchasing behavior of participants in this virtual shopping experiment (for overall purchases or tobacco-specific purchases) based on whether we told them that they would receive the products they selected (ie, using deception) or not. This finding suggests that deception does not affect the products that participants select in a virtual store.

No previous study, to our knowledge, has examined the impact of deception on purchasing behavior in a virtual store experiment. Our results suggest that deception may not make the virtual store more believable, that is, more likely to reflect consumer preferences (the products that consumers actually want to purchase). Our results are consistent with several research studies that found no differences between experimental conditions that contained deception and those that did not. Barrera and Simpson [15] conducted a classic prisoner’s dilemma study with sociology students at a large university. Participants in the treatment condition were told that they had a partner who was involved in the decision-making task but were afterward told that no partner existed, while participants in the control condition were assigned a real partner. The researchers found no difference in the behavior of the participants in the prisoner’s dilemma task across conditions. Similarly, a review found that, in previous studies involving deception, there was no difference in the experimental behavior of participants who were and were not deceived. Only when participants were explicitly told before the study that they would be deceived or were intentionally made suspicious of the purpose of the study by experimenters did their behavior differ [16].

Although the literature has not examined the role of deception in virtual stores, existing research has found that virtual stores provide valid information on actual shopping behavior. Using a consumer panel recruited by phone in the Netherlands, van Herpen et al [1] randomly assigned participants to a physical store, a virtual store, or to view screenshots of the virtual store, then compared the products they selected for purchase across the three conditions. For all conditions, participants were reimbursed with gift certificates (E van Herpen, electronic communication, February 2018). Researchers confirmed that, after the experiment, all participants in the physical store condition used their gift certificates to purchase (in the same physical store) the products that they had selected during the experiment. They did not compare the products that participants in the virtual store or screenshot conditions selected to those same participants’ later purchases in real stores. van Herpen et al [1] found that participants in the virtual store and screenshot conditions purchased more products and a greater variety of products than participants in the physical store condition. However, purchases of participants in the virtual store condition more closely resembled the purchases of participants in the physical store condition than they resembled the purchases of participants in the screenshot condition. Waterlander et al [5] recruited adults in New Zealand using print and online advertising and found some differences in the amount of money spent on food groups between the virtual store and participant-provided receipts. However, the 4 food groups with the highest relative expenditures were the same for the virtual stores and receipts. Taken together, these results suggest that virtual stores are likely to be a valid method of assessing shopping behavior.

### Limitations

Based on the findings of this analysis alone, we cannot definitively conclude that deception is not needed in virtual store research. First, it is possible that unmeasured confounders explain the nonsignificant difference in shopping behavior by condition; however, randomization lessens this possibility. Second, we cannot be sure that deception was successful. Although we know that most participants in the deception condition understood that they would receive the products (or some of them) and that they might get a gift card instead of the products, it remains unclear how many of them believed they would receive the products (as opposed to the gift card). One potential explanation for the lack of differences in shopping behavior by condition is that neither group believed that they would receive the products they selected. It also remains unclear whether respondents’ in-store shopping behavior would have been different had we promised to give them the products they selected immediately after they completed the experiment (which was not possible because the study was administered online), as opposed to saying we would mail them the products at a later date. Given these limitations, the results of this analysis should be interpreted with caution.

### Conclusion

The purchasing behavior of virtual shoppers did not vary based on whether or not they were told that they would receive the products they selected in the virtual store. The results of this study suggest that deception about the receipt of goods following a virtual shopping task is unlikely to affect consumer behavior in virtual stores.

## Appendix

Multimedia Appendix 1 Screenshots of the iShoppe virtual convenience store.

Multimedia Appendix 2 Consolidated Standards of Reporting Trials (CONSORT) flow diagram.

Multimedia Appendix 3 CONSORT-EHEALTH checklist (V 1.6.1).

Multimedia Appendix 4 Demographic characteristics and tobacco use behavior by experimental group among participants who completed a virtual shopping experiment (n=398).

## Abbreviations

RTI: Research Triangle Institute
