# Supplementary material for: Deception and Shopping Behavior Among Current Cigarette Smokers: A Web-Based, Randomized Virtual Shopping Experiment
Source: JMIR Res Protoc. 2018 Jun 29;7(6):e10468. doi: 10.2196/10468 (PMC6045792; doi:10.2196/10468)

MULTIMEDIA APPENDIX 1

Screenshots of the iShoppe^{TM} Virtual Convenience Store

Figure 1. Screenshot of iShoppe^{TM} virtual store instructions

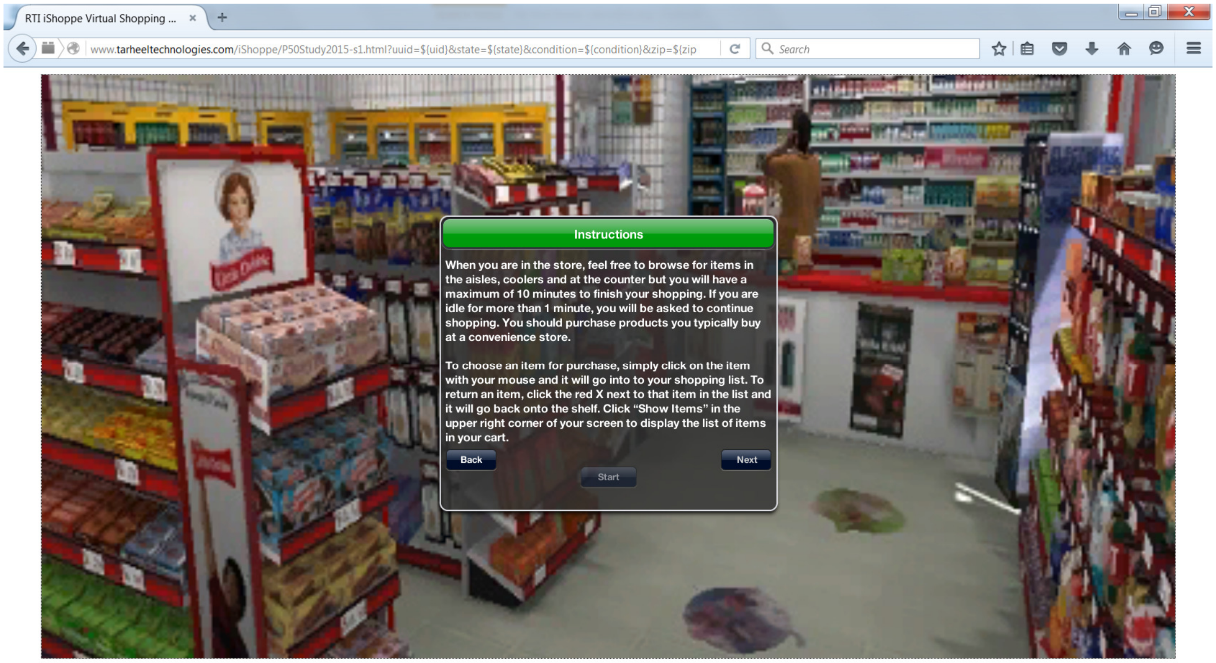

Figure 2. Screenshot of iShoppe^{TM} virtual store entrance

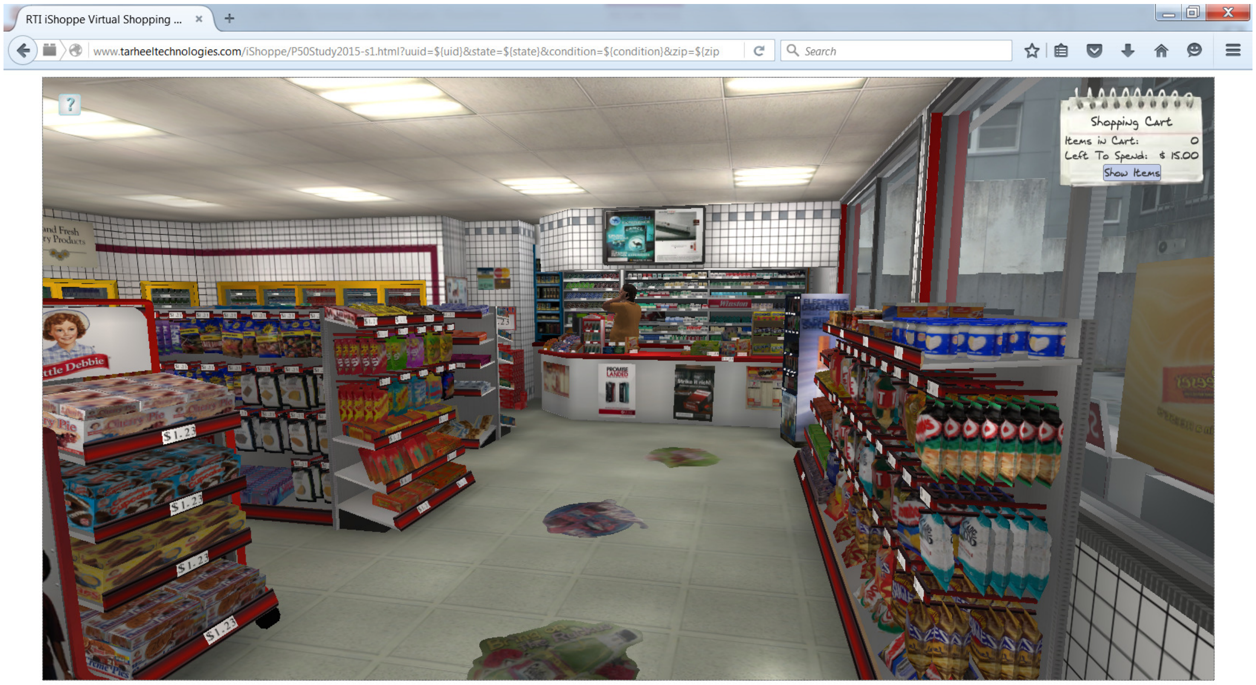

Figure 3. Screenshot of iShoppe™ virtual store checkout counter.

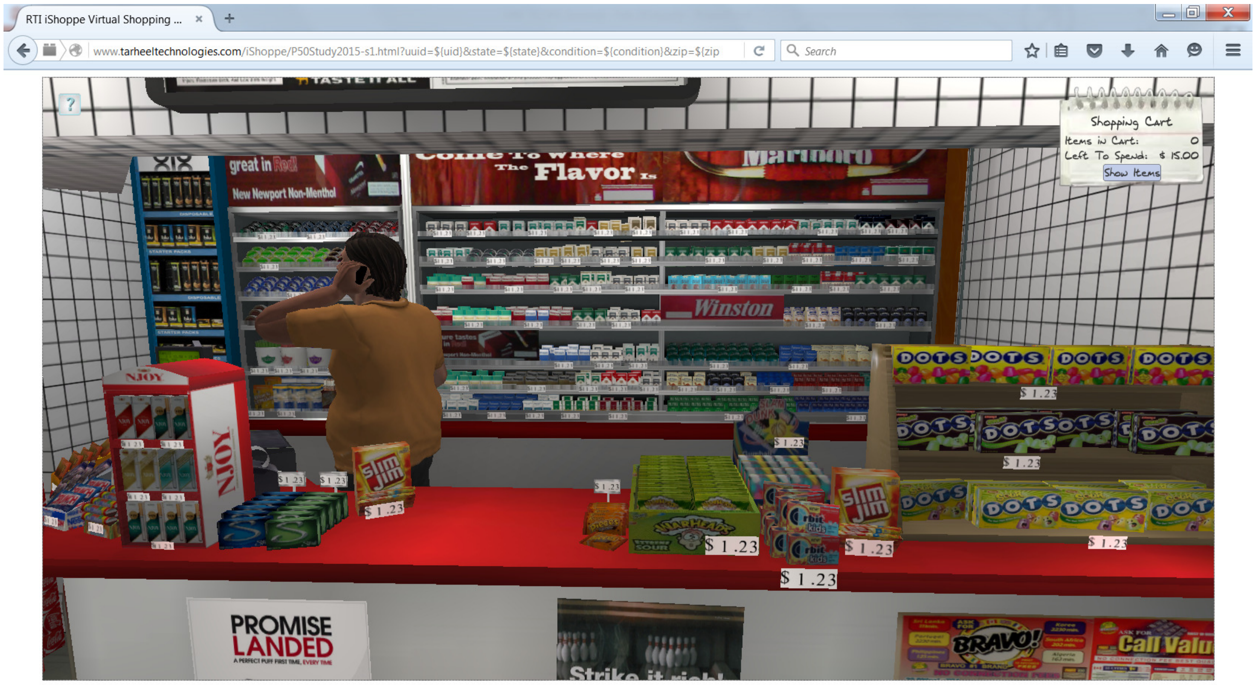

Supplement: Multimedia Appendix 1 [file resprot_v7i6e10468_app1.pdf]
