# Supplementary figures and images for: Deception and Shopping Behavior Among Current Cigarette Smokers: A Web-Based, Randomized Virtual Shopping Experiment
Source: JMIR Res Protoc. 2018 Jun 29;7(6):e10468. doi: 10.2196/10468 (PMC6045792; doi:10.2196/10468)

## MULTIMEDIA APPENDIX 1

### CONSORT Flow Diagram

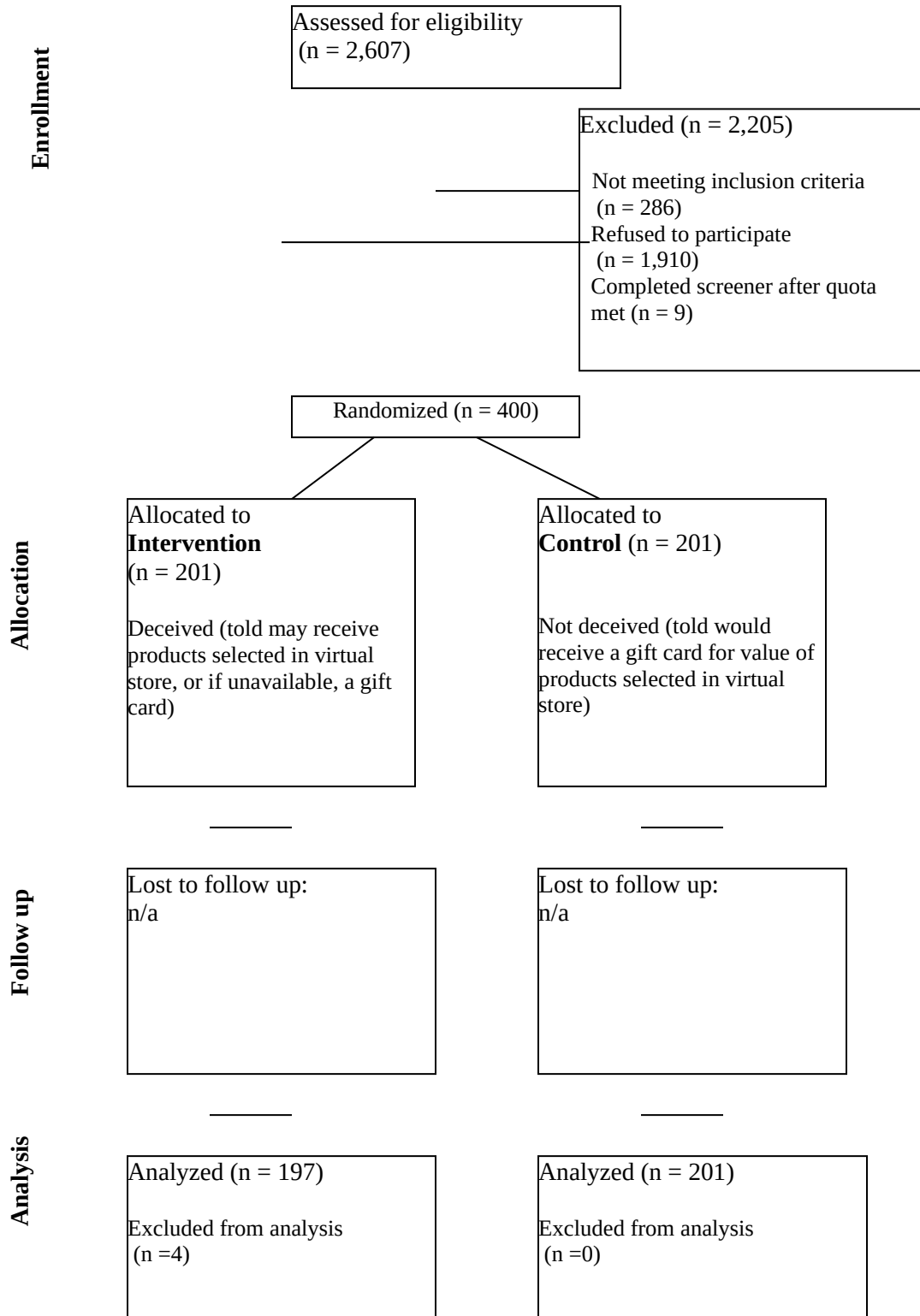

Supplement: Multimedia Appendix 2 [file resprot_v7i6e10468_app2.pdf]
