# Supplementary material for: Deception and Shopping Behavior Among Current Cigarette Smokers: A Web-Based, Randomized Virtual Shopping Experiment
Source: JMIR Res Protoc. 2018 Jun 29;7(6):e10468. doi: 10.2196/10468 (PMC6045792; doi:10.2196/10468)
Supplement: Multimedia Appendix 4 [file resprot_v7i6e10468_app4.pdf]

MULTIMEDIA APPENDIX 4

Demographic Characteristics and Tobacco Use Behavior by Experimental Group Among Participants Who Completed a Virtual Shopping Experiment (n = 398)

		**Experimental Group**		
	**Total**<br>**n = 398**<br>**n (%)**	**Control**[a]<br>**n = 201**<br>**n (%)**	**Intervention**[a]<br>**n = 197**<br>**n (%)**	***P*-value**
**Demographics**				
Age				
18–34	141 (35.4)	76 (37.8)	65 (33.0)	.48
35–54	182 (45.7)	91 (45.3)	91 (46.2)	
55+	75 (18.8)	34 (16.9)	41 (20.8)	
Female	187 (47.0)	84 (41.8)	103 (52.3)	.04
Race				
White	296 (74.4)	152 (75.6)	144 (73.1)	.84
Black	20 (5.0)	11 (5.5)	9 (4.6)	
Hispanic	59 (14.8)	27 (13.4)	32 (16.2)	
Other	23 (5.8)	11 (5.5)	12 (6.1)	
Education				
Some high school or less	5 (1.3)	2 (1.00)	3 (1.5)	.15
High school grad/GED	58 (14.6)	26 (12.9)	32 (16.2)	
Some college	130 (32.7)	58 (28.9)	72 (36.6)	
College grad /postgrad/professional	205 (51.5)	115 (57.2)	90 (45.7)	
Annual household income				
<$25,000	64 (16.2)	29 (14.5)	35 (18.0)	.59

	Total	Experimental Group		
		Control[a]	Intervention[a]	
	n = 398<br>n (%)	n = 201<br>n (%)	n = 197<br>n (%)	*P*-value
$25,000–$49,999	86 (21.8)	45 (22.5)	41 (21.0)	
$50,000–$74,999	91 (23.0)	43 (21.5)	48 (24.6)	
$75,000+	154 (39.0)	83 (41.5)	71 (36.4)	
**Tobacco Use**				
Days smoked				
1 or 2 days	7 (1.8)	4 (2.0)	3 (1.5)	.55
3–5 days	14 (3.5)	10 (5.0)	4 (2.0)	
6–9 days	30 (7.5)	13 (6.5)	17 (8.6)	
10–19 days	41 (10.3)	23 (11.4)	18 (9.1)	
20–29 days	50 (12.6)	24 (11.9)	26 (13.2)	
All 30 days	255 (64.1)	126 (62.7)	129 (65.5)	
Ever smoked daily	382 (96.2)	193 (96.5)	189 (95.9)	.77
Last smoked				
Within the last one hour	194 (48.7)	2 (45.8)	102 (51.8)	.71
1–2 hours ago	89 (22.4)	45 (22.4)	44 (22.3)	
2–5 hours ago	45 (11.3)	24 (11.9)	21 (10.7)	
5–10 hours ago	25 (6.3)	16 (8.0)	9 (4.6)	
Within the past day	29 (7.3)	15 (7.5)	14 (7.1)	
2 days ago or longer	16 (4.0)	9 (4.5)	7 (3.6)	
How soon smoke after waking				
Within 5 minutes	84 (21.1)	38 (18.9)	46 (23.4)	.28

	Total	Experimental Group		
		**Control[a]**	**Intervention[a]**	
	**n = 398**<br>**n (%)**	**n = 201**<br>**n (%)**	**n = 197**<br>**n (%)**	***P*-value**
6–30 minutes	175 (44.0)	98 (48.8)	77 (39.1)	
31–60 minutes	75 (18.8)	35 (17.4)	40 (20.3)	
After 60 minutes	64 (16.1)	30 (14.9)	34 (17.3)	
Now chew tobacco	87 (21.9)	41 (20.5)	46 (23.4)	.49
Now use cigars, cigarillos, little cigars	135 (33.9)	66 (32.8)	69 (35.0)	.65
Stopped smoking 1+ day past 12 months	156 (39.2)	82 (40.8)	74 (37.6)	.51
Want to quit smoking				
Not at all	49 (12.3)	18 (9.0)	31 (15.7)	.14
A little	110 (27.6)	53 (26.4)	57 (28.9)	
Somewhat	120 (30.2)	66 (32.8)	54 (27.4)	
A lot	119 (29.9)	64 (31.8)	55 (27.9)	
Plan to stop smoking in next 30 days	272 (68.3)	133 (67.9)	139 (71.7)	.42
Understood that they would receive some or all products selected or gift card	152 (87.4)	N/A	152 (77.2)	-
	**Mean (SD)**	**Mean (SD)**	**Mean (SD)**	**p-value**
Mean cigs per day	16.0 (14.1)	16.6 (15.6)	15.5 (12.3)	.23

[a] Participants in the intervention (deception) condition were told that they would receive the products that they selected in the virtual store by mail (or the cash equivalent if the products were unavailable). Participants in the control (no deception) condition were told they would receive the cash equivalent of the products they selected.
